# Risk Model for Prostate Cancer Using Environmental and Genetic Factors in the Spanish Multi-Case-Control (MCC) Study

**DOI:** 10.1038/s41598-017-09386-9

**Published:** 2017-08-21

**Authors:** Inés Gómez-Acebo, Trinidad Dierssen-Sotos, Pablo Fernandez-Navarro, Camilo Palazuelos, Víctor Moreno, Nuria Aragonés, Gemma Castaño-Vinyals, Jose J. Jiménez-Monleón, Jose Luis Ruiz-Cerdá, Beatriz Pérez-Gómez, José Manuel Ruiz-Dominguez, Jessica Alonso Molero, Marina Pollán, Manolis Kogevinas, Javier Llorca

**Affiliations:** 1 CIBER Epidemiologia y Salud Publica (CIBERESP), Madrid, Spain; 2 University of Cantabria – IDIVAL, Santander, Spain; 3 Cancer and Environmental Epidemiology Unit, National Center for Epidemiology, Carlos III Institute of Health, Madrid, Spain; 4 Cancer Prevention and Control Program, Catalan Institute of Oncology-IDIBELL, and University of Barcelona, Barcelona, Spain; 5 Cancer Epidemiology Research Group. Oncology and Hematology Area. IIS Puerta de Hierro (IDIPHIM), Madrid, Spain; 6 ISGlobal, Centre for Research in Environmental Epidemiology (CREAL), Barcelona, Spain; 7 Universitat Pompeu Fabra (UPF), Barcelona, Spain; 8 IMIM (Hospital del Mar Medical Research Institute), Barcelona, Spain; 9 Instituto de Investigación Biosanitaria de Granada (ibs.GRANADA), Hospitales, Granada, Spain; 10 Urology Department, La Fe University Hospital, Avinguda de Fernando Abril Martorell 106, 46026 Valencia, Spain; 11 Urology Department, Hospital Germans Trias i Pujol, Carretera de Canyet, S/N, 08916 Badalona, Barcelona Spain

## Abstract

Prostate cancer (PCa) is the second most common cancer among men worldwide. Its etiology remains largely unknown compared to other common cancers. We have developed a risk stratification model combining environmental factors with family history and genetic susceptibility. 818 PCa cases and 1,006 healthy controls were compared. Subjects were interviewed on major lifestyle factors and family history. Fifty-six PCa susceptibility SNPs were genotyped. Risk models based on logistic regression were developed to combine environmental factors, family history and a genetic risk score. In the whole model, compared with subjects with low risk (reference category, decile 1), those carrying an intermediate risk (decile 5) had a 265% increase in PCa risk (OR = 3.65, 95% CI 2.26 to 5.91). The genetic risk score had an area under the ROC curve (AUROC) of 0.66 (95% CI 0.63 to 0.68). When adding the environmental score and family history to the genetic risk score, the AUROC increased by 0.05, reaching 0.71 (95% CI 0.69 to 0.74). Genetic susceptibility has a stronger risk value of the prediction that modifiable risk factors. While the added value of each SNP is small, the combination of 56 SNPs adds to the predictive ability of the risk model.

## Introduction

Prostate cancer (PCa) is, after lung cancer, the second most common cancer among men worldwide. The incidence of prostate cancer is increasing in all countries, especially in western countries^1^, on the one hand, due to the population aging and increased exposure to environmental risk factors, and secondly, due to the generalization of screening and improved diagnostic techniques. This, together with decreasing mortality from the disease, has contributed to the increase in prevalence. In Spain in 2015, 27,853 new cases and 5,481 deaths are estimated^1^.

Despite its considerable impact, the etiology of prostate cancer remains largely unknown compared to other common cancers. The only well-established risk factors for PCa are advanced age, family history, and ethnicity^2–6^. Other factors such as diet (a high-calorie or high-fat diet, and high level of cholesterol, high consumption of meat products, fruits, fish, milk, dairy foods, dietary calcium and dietary vitamin D, and spirits), high BMI, weight, waist circumference, physical inactivity and inflammation have been proposed as risk factors for PCa^7–13^, but their role in prostate cancer etiology remains unclear.

Hereditary component accounts for up to 42% of the PCa risk, including individual and combined effects of rare highly penetrant genes, common weak penetrant genes, and genes that act in concert with others^14–16^. In fact, it has been shown that PCa is the neoplasia with a higher hereditary component^15^. However, to identify genetic variants associated with the disease is not being an easy task. To establish which genetic factors play a role in the development and progression of prostate cancer could help to determine who is at greatest risk, leading to early detection and/or developing new therapeutic treatments. Studies of genome-wide association (GWAS) are being designed with increasing statistical power in order to identify genetic variants associated with complex human diseases^17^. GWAS through massive genotyping case-control comparison have allowed for the discovery of dozens of SNPs associated with diseases other than genetic etiologies. Since 2006 more than 76 SNPs related with PCa risk have been cataloged, explaining 30% of the heritability of PCa^18^. Each separate allele confers a small individual risk (odds ratios [OR] between 1.06 to 1.79 per allele)^18–20^. The detection of these risk alleles in an individual, together with the value of prostate specific antigen (PSA), family history and environmental factors, could increase the specificity and sensitivity of PCa diagnostic tests. In this study, we have developed a risk stratification model that combines environmental factors with family history and genetic susceptibility. We evaluated the relative contribution of these factors and the utility of the model for risk stratification and public health intervention.

## Methods

### Study design and population

The Multi Case-Control (MCC-Spain) study is a population based case-control study of common tumors in Spain and has been described elsewhere^21^. It has been carried out in 23 hospitals and primary care centers in 12 Spanish provinces. The recruitment includes incident cases of colorectal, breast, stomach and prostate cancer or chronic lymphocytic leukemia diagnosed between September 1st, 2008 and December 31st, 2013. All cases were incident and pathology-confirmed, aged between 20 and 85 years old and resident within the influence area of the hospital at least 6 months prior to recruitment.

In this paper, 818 cases of prostate cancer (ICD-10: C61, D07.5) and 1,006 frequency-matched controls with genotype data were considered. Controls were men whit no prior history of prostate cancer living in the same catchment area as cases; they were randomly selected from the rosters of General Practitioners at the Primary Health Centers. Controls were frequency-matched to cases by 5-year age groups and study area. Among these cases and controls, there were only two Arabic individuals (one case and one control); the rest of the participants were White/Caucasian. Response rates varied between centers and were on average 74% among cases (range 47–94%) and 54% (range 30–94%) among controls with valid telephone numbers in the PHC rosters.

All procedures performed in studies involving human participants were in accordance with the ethical standards of the institutional and/or national research committee, and with the 1964 Helsinki Declaration and its later amendments or comparable ethical standards. The protocol of MCC-Spain was approved by each of the ethics committees of the participating institutions. The specific study reported here was approved by the Ethical Committee of Clinical Research of Asturias, Barcelona, Cantabria, Girona, Gipuzkoa, Huelva, León, Madrid, Navarra and Valencia). Informed consent was obtained from all individual participants included in the study.

### Data collection

Participants were interviewed face-to-face by trained interviewers who used a comprehensive epidemiological questionnaire that assessed socio-demographic information, personal and family history of cancer, anthropometric data, smoking habits and physical activity habits, water consumption, reproductive and medical history and medication/drug use. Diet was assessed with the use of a validated semi-quantitative Spanish Food Frequency questionnaire (FFQ), which was modified to include regional products^22^. The FFQ included 140 food items, and assessed usual dietary intake for the previous year. Blood samples were obtained following the study protocol.

Participant’s weight was self-reported, as estimated one year before diagnosis for cases and for controls. Their body mass index (BMI) was calculated from self-reported weight and height noted the year prior to the diagnosis of cases and one year before to the interview for controls. Similar estimates provided total energy consumption. Physical activity was recorded for all jobs and also recreational physical exercise.

### Modifiable and non modifiable risk factor score

Only variables previously reported with PCa have been considered for the development of risk models^23^. The variables considered were: family history of PCa (none versus first or second or third-degree); cigarette smoking, grouped into non-smokers and smokers (including former and current); average alcohol consumption between 30 to 40 years old (in standard units of alcohol, SUA) and spirits categorized in tertiles. To ensure that the exposures were prior to the effect, it was decided to collect alcohol consumption in the past (30–40 year-old). It appears that some prostate cancers can pass through a latency period of up to 15 to 20 years, during which the disease is histologically present but has not yet come to attention. However, we do believe that trying to record dietary in the same way would be strongly biased as it is not expected participants could complete a diet questionnaire after such a long period. Alcohol consumption during the previous year has been collected and the results are very similar to those of the past. BMI (calculated with the weight reported one year prior to the interview for controls and one year prior to the diagnosis for cases). It was categorized according to The World Health Organization criteria: underweight, normal weight and overweight (<30 kg/m2) versus obese (≥30 kg/m2); weight and waist circumference were categorized in tertiles. Average physical exercise, measured from self-reported leisure-time activity performed in the past 10 years was used to estimate the Metabolic Equivalent of Task (MET) per hour per week, calculated using the Ainsworth’s compendium of physical activities^24^, and categorized according to the WHO’s classification in light/moderate/vigorous (using thresholds: 3 and 6 METs)^25^. Red meat consumption, that included meat from mammals (cattle, ox, veal, beef, pork, etc.), from game (duck, pheasant, etc.), offal (liver, brains, etc.), cured meat (ham, bacon, etc.) and processed meat (hot dogs, sausages, meat balls, etc.). High intake of red meat was considered eating ≥65 g/day (it is average meat consumption among controls); vegetables and fruit separately, classified as low or high intake using 200 g/day as cut-off, following the strategy to reduce the prevalence of noncommunicable diseases launched by WHO in 2004, which includes five or more servings of fruit and/or vegetables per day (≥400 g/day)^26^. Fish, milk, dairy foods (including milk, yogurt and cheese), dietary calcium and vitamin D were categorized in tert too. We have introduced categorization into tertile in order to avoid making specific assumptions about the shape of their relationship with the outcome and minimize the influence of outliers.

### Genotyping and SNP selection for the genetic risk scores

The genotyping was performed using the Infinium Human Exome BeadChip (Illumina, San Diego, USA) that includes >200 000 coding markers plus 5000 additional custom SNPs selected from previous GWAS studies or in genes of interest.

Genomebrowser^27^ was used to identify those genetic variants associated with PCa through genome-wide association studies (GWAS) in which the “*reported trait*” was prostate cancer with a p-value threshold of 5 · 10^−8^, apparent in the population with European ancestry population (initial or replication sample). For this analysis, we could include 62 of these PCa susceptibility variants: 51 were included in our genotyping array, and another 11 variants, not included in our Exome BeadChip, were also estimated through a “proxy”, considered as such a SNP genotyped in our array that was in linkage disequilibrium (LD) (r^2^ > 0.8) with them according with 1000 Genomes data. For proxy search, we used “SNP Annotation and Proxy Search” (SNAP)^28, 29^ with the following inputs: “1000 Genomes Pilot 1” as SNP data set, CEU population as “panel” chosen, 0.8 as “r^2^ threshold”, and a distance of 500 kilobases between the query SNP and the proxy SNP. In each case, we selected the proxy SNP with the highest r^2^ that was genotyped in our array.

Finally, six of the 62 SNPs had to be eliminated because they were in linkage disequilibrium with other snps (r^2^ > 0.8). In this way, we finally included 56 SNPs in the analysis. No SNP was discarded according to Hardy-Weinberg equilibrium (HWE) as no SNP showed statistical significance (p < 10^−4^) in the HWE test. The complete list of SNPs is detailed in Supplementary Table 1.

### Statistical analysis

Multivariate logistic regression models were used to build risk models. All models were adjusted to a propensity score^30^ to avoid bias related to differences in case and control selection frequencies. The propensity score model was constructed as the individual prediction (in logit scale) of a logistic regression in which case/control status was modeled with age, level of education, recruiting center, and the first 3 principal components of genetic ancestry obtained from GWAS genotyping data. The propensity score was added as a continuous variable to adjust the risk models.

An environmental risk score (ERS) was built and included all covariates with a p value ≤ 0.20 and that can be modified (diabetes, weigh, BMI, alcohol and red meat). This score was built by logistic regression, by adding the estimated beta coefficients of the above indicated risk factors. The results are presented as odds ratios (OR) with 95% confidence intervals (CI) by deciles of the ERS. All p-values given are two tails. Family history of PCa was not considered in this environmental score since it is not modifiable, and its effect was assessed as a separate factor.

To assess genetic susceptibility, an additive genetic risk score was elaborated. Each SNP was coded as 0, 1 or 2 copies of the sample risk allele, defined as the allele with highest PCa risk, except for the SNP rs5945572 in chromosome X that was coded 0 or 1.

We elaborated a genetic risk score (GRS) by adding the beta coefficients obtained in the logistic regression analysis. We avoided using published weights, which could be overestimated.

Results are reported as odds ratios (OR) with 95% confidence intervals (CI) by decile of the GRS. All reported p-values are two-tailed.

The predictive accuracy of models was assessed with the area under the ROC curve (AUROC), adjusted for the propensity score. Weights were proportional to the number of cases in each decile. A 95% CI was calculated for the AUROC using bootstrapping techniques for internal validation, using 1000 replications. The bootstrap principle is to sample the empirical distribution from which the data originated (i.e. sampling with replacement of observed data)^31^. The performance estimate were corrected by optimism^32^.

For analyzing whether a score adds or not to the predictive ability of another score, we estimated the net reclassification improvement (NRI), the integrated discrimination improvement (IMI) and the improvement in the AUROC. The goal for a score is to classify prostate cancer cases and controls adequately; when two scores -say ERS and GRS- are compared, GRS would classify some cases better than ERS and some others worse; likewise, GRS would classify some controls better and some others worse. NRI^33^ is the net sum of classifying cases and controls better using GRS rather than ERS:

It is noteworthy that NRI can only be applied when both scores are nested one in each other, as it is the case between ERS and GRS, because the risk of prostate cancer is the sum of genetic and environmental risk factors. In this article, we use the continuous version of NRI^34^.

While NRI measures the improvement in classification, IMI estimates the improvement in predicted probability. A better score is expected to predict higher probabilities in cases and lower probabilities in controls than a worse score. Therefore, IMI is defined as^33^:

To estimate the potential public health impact of the environmental and genetic risk scores, we applied the estimated relative risks (ORs) to average population PCa incidence estimations published by The International Agency for Research on Cancer (IARC). Data was extracted from GLOBOCAN 2012: estimated cancer incidence, mortality and prevalence worldwide in 2012^35^. Average age specific incidence rates for the Spanish population were projected according to combinations of environmental and genetic risk scores to define risk strata. For these estimates, the published rates were multiplied by the ORs estimated from out risk models. We used the average number of risk factors and risk alleles in the population as reference category for these calculations.

Statistical analysis was carried out using the package Stata 14/SE (StataCorp, College Station, Tx, US).

## Results

### Population description and modifiable risk factor score

A description of the 818 cases and 1006 controls included in this study is provided in Table 1. Variables have been coded with the lower PCa risk category as reference to simplify comparisons of effect and elaboration of risk scores. Family history of prostate cancer is a well-known risk factor for prostate cancer. In our study, having a family member with this tumor multiplied the risk of prostate cancer by 3.29 (OR = 3.29, 95% CI = 2.44 to 4.43). Among the modifiable factors, Diabetes behaved as a protective factor against prostate cancer in those who were either untreated (OR = 0.41, 95% CI = 0.22 to 0.77), or in those whose treatment was with oral antidiabetic agents (OR = 0.69, 95% CI = 0.52 to 0.92). While consumption of alcohol in the third tertile and high consumption of red meat behaved as a risk factor for PCa (OR = 1.29, 95% CI = 1.01 to 1.65 and OR = 1.35, 95% CI = 1.10 to 1.65 respectively). Regarding the aggressiveness of the tumor, only 14% of the prostate cancers studied were high risk (Gleason score ≥8), compared to 45% that were low grade (Gleason ≤6).

**Table 1:** Characteristics of the MCC-Spain study participants.

Characteristic	Control		Case		Crude OR	95% CI
	n	%	n	%		
**Age**						
<65 years	410	40.76	342	41.81	1.00	
≥65 years	596	59.24	476	58.19	0.95	0.79–1.15
**Family History of PCa**						
No	930	92.72	649	79.73	1.00	
Yes	**73**	**7**.**28**	**165**	**20**.**27**	**3**.**29**	**2**.**44–4**.**43**
**Diabetes**						
No	783	77.99	688	84.42	1.00	
Yes, without pharmacological treatment	**37**	**3**.**69**	**14**	**1**.**72**	**0**.**41**	**0**.**22–0**.**77**
Yes, with oral antidiabetic treatment	**143**	**14**.**24**	**86**	**10**.**55**	**0**.**69**	**0**.**52–0**.**92**
Yes, treated with insulin	25	2.49	12	1.47	0.58	0.29–1.18
Yes, both treatments (oral + insulin)	16	1.59	15	1.84	1.02	0.50–2.08
**Hypertension**						
No	498	49.65	448	54.97	1.00	
Yes, without pharmacological treatment	38	3.79	27	3.31	0.79	0.47–1.32
Yes, with pharmacological treatment	**467**	**46**.**56**	**340**	**41**.**72**	**0**.**80**	**0**.**66–0**.**96**
**Smoking**						
Non-smoker	275	27.34	240	29.34	1.00	
Former/Current smoker	731	72.66	578	70.66	0.91	0.74–1.13
**Alcohol**						
Tertile 1	310	35.71	217	30.52	1.00	
Tertile 2	**274**	**31**.**57**	**253**	**35**.**58**	**1**.**29**	**1**.**01–1**.**65**
Tertile 3	284	32.72	241	33.90	1.17	0.91–1.50
**Spirits** (**g/d**)						
Tertile 1	534	59.87	449	61.42	1.00	
Tertile 2	175	19.62	120	16.42	0.82	0.63–1.07
Tertile 3	183	20.52	162	22.16	1.01	0.79–1.30
**Body Mass Index one year before**						
Normal (18.5 to 24.9 kg/m2)	240	23.86	210	25.67	1.00	
Overweight (25.0–29.9 kg/m2),	528	52.49	424	51.83	0.90	0.72–1.13
Obese (≥30 kg/m^2^)	235	23.36	182	22.25	0.83	0.63–1.09
**Weight**						
Tertile 1	332	33.33	283	34.72	1.00	
Tertile 2	332	33.33	291	35.71	0.99	0.79–1.24
Tertile 3	332	33.33	241	29.57	0.85	0.67–1.07
**Waist circumference**						
Tertile 1	362	36.49	277	37.48	1.00	
Tertile 2	322	32.46	247	33.42	1.00	0.79–1.26
Tertile 3	308	31.05	215	29.09	0.91	0.71–1.15
**Physical Activity in Leisure Time**						
Moderate/vigorous (≥6 METs)	611	60.74	519	63.45	1.00	
Light (<3 METs)	395	39.26	299	36.55	0.90	0.74–1.10
**Vegetables**						
>200 g/day	257	29.61	226	31.79	1.00	
≤200 g/day	611	70.39	485	68.21	0.91	0.73–1.13
**Fruits**						
>200 g/day	611	70.39	519	73.00	1.00	
≤200 g/day	257	29.61	192	27.00	0.84	0.67–1.05
**Fish**						
Tertile 3	290	32.51	251	34.34	1.00	
Tertile 2	296	33.18	242	33.11	0.95	0.75–1.21
Tertile 1	306	34.30	238	32.56	0.90	0.71–1.15
**Red Meat**						
≤65 g/day	440	50.69	309	43.46	1.00	
>65 g/day	428	49.31	402	56.54	**1**.**35**	**1**.**10–1**.**65**
**Milk**						
Tertile 1	336	37.67	281	38.44	1.00	
Tertile 2	249	27.91	219	29.96	1.08	0.85–1.38
Tertile 3	307	34.42	231	31.60	0.97	0.77–1.23
Dairy foods*						
Tertile 1	293	32.85	248	33.93	1.00	
Tertile 2	302	33.86	241	32.97	0.96	0.75–1.22
Tertile 3	297	33.30	242	33.11	1.04	0.81–1.32
**Dietary calcium** (**mg/d**)						
Tertile 1	293	32.85	248	33.93	1.00	
Tertile 2	304	34.08	237	32.42	0.94	0.74–1.20
Tertile 3	295	33.07	246	33.65	1.04	0.81–1.32
**Dietary vitamin D**						
Tertile 1	293	32.85	248	33.93	1.00	
Tertile 2	295	33.07	246	33.65	0.99	0.78–1.26
Tertile 3	304	34.08	237	32.42	0.96	0.73–1.18

*Dairy foods include milk, yogurt and cheese.

Associations with *P* < 0.05 are shown in bold.

Table 2 shows the multivariate risk factors associated with PCa. Family history of PCa was strongly associated to PCa (adjusted OR 3.32, 95% CI 2.34 to 4.71). Regarding environmental risk factors, consumption of red meat higher than 65 grams a day (adjusted OR 1.28, 95% CI 1.02 to 1.61) and the second tertile of alcohol (adjusted OR 1.46, 95% CI 1.10 to 1.93) contributed to PCa risk, while diabetes contributed to reduce the risk of PCa (OR = 0.58, 95% CI 0.43 to 0.79).The environmental score, calculated by adding the estimated beta coefficients of the risk factors listed above, also reached statistical significance (OR = 2.47; 95% CI 1.62 to 3.76). Supplementary Figure 1 shows the distribution of the environmental score for cases and controls, and the estimated risk of PCa according to the deciles of the estimated beta coefficients of the risk factors.

**Table 2:** Multivariate-adjusted risk factors associated with PCa.

			Adjusted OR^a^	CI 95%
Genetic Risk Score	**GRS**	(**per unit**)	**2**.**05**	**1**.**79–2**.**36**
Family History of PCa			**3**.**32**	**2**.**34–4**.**71**
Environmental risk score	**Diabetes**	**yes**	**0**.**58**	**0**.**43–0**.**79**
	**Weight**	**Tertile 2** (**74–83 Kg**)	0.94	0.69–1.23
		**Tertile 3** (**84–135 Kg**)	0.70	0.48–1.03
	**BMI**	**Overweight** (**25**.**0 to 29**.**9 kg/m2**)	1.03	0.74–1.42
		**Obese** (**≥30 kg/m** ^**2**^)	1.26	0.80–1.99
	**Alcohol**	**Tertile 2** (**7**.**73–20**.**77 g/d**)	**1**.**46**	**1**.**10–1**.**93**
		**Tertile 3** (**≥20**.**8 g/d**)	1.19	0.90–1.57
	**Red meat**	>**65 g/day**	**1**.**28**	**1**.**02–1**.**61**
	**ERS**	(**per unit**)	**2**.**47**	**1**.**62–3**.**76**

GRS: genetic risk score; ERS: environmental risk score; BMI: body mass index.

aAll variables are adjusted by propensity score and all the variables shown in the table.

Associations with P < 0.05 are shown in bold.

### Genetic risk score

**Figure 1:**
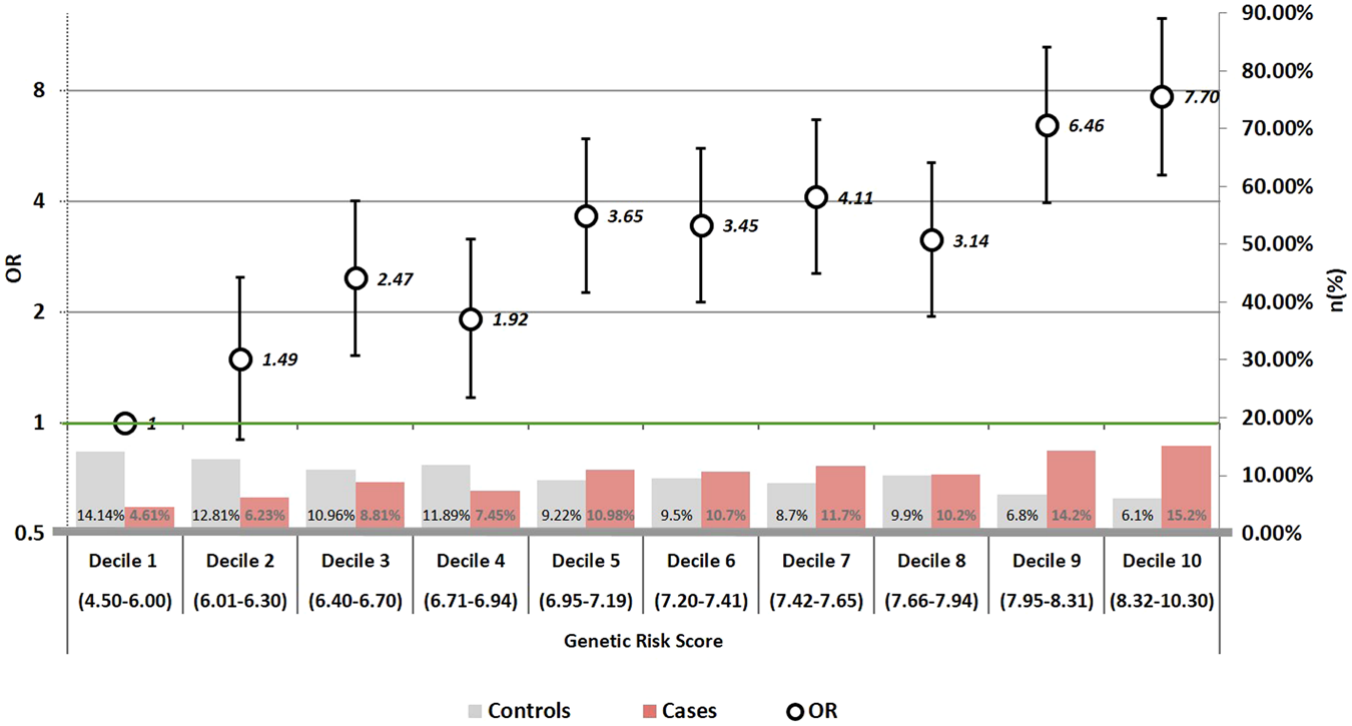
Distribution of prostate cancer cases and controls, and odds ratios for each decile of the genetic risk score. The left axis scale indicates the OR for prostate cancer according to deciles of points in the genetic score. The decile 1 (4.5–6.0 points) has been selected as reference category (OR = 1). The right axis scale indicates the proportion of cases and controls shown in bars for each decile. Parentheses include the points in the score.

Out of 56 GWAS SNPs analyzed, only 26 were statistically associated with PCa in our data. There were eight (rs2660753, rs7629490, rs9364554, rs7758229, rs6465657, rs4242382, rs4430796, rs17632542) for which the association is significant only for the heterozygous genotype. (Supplementary Figure 1 and 2). The less frequent allele was protective in 9 SNPs, and increased the risk in the remaining 9 SNPs. The genetic risk score (GRS) built by adding the estimated beta coefficients had an average of 7.5 in cases and 6.9 in controls (Fig. 1); the GRS was significantly associated with PCa, with an average per unit OR of 2.05 (95% CI 1.79 to 2.36).

As shown in Fig. 1, the increase in risk per GRS decile was almost linear, indicating the independent additive contribution of each beta coefficients to the genetic risk score. Compared to subjects scoring 4.5–6.00 (reference category, decile 1), those scoring 6.95–7.19 (decile 5) had a 265% increase in PCa risk (OR = 3.65, 95% CI 2.26 to 5.91), while PCa risk in subjects with scoring 8.32–10.30 (decile 10) was 7 times higher than that of the reference category (OR = 7.70, 95% CI 4.72 to 12.58). The GRS was independent of modifiable environmental variables and no significant interactions were observed between the genetic risk score and age or any of the environmental variables included in the multivariate model. The Supplementary Figure 1 shows the same figure but for the environmental score. Compared with decile 1 (reference), statistical significance was only reached from the decile 7 on, although with less magnitude than in the genetic risk score.

### Estimating the potential public health impact of a risk model to stratify screening or modify risk factors in the average Spanish risk population

**Figure 2:**
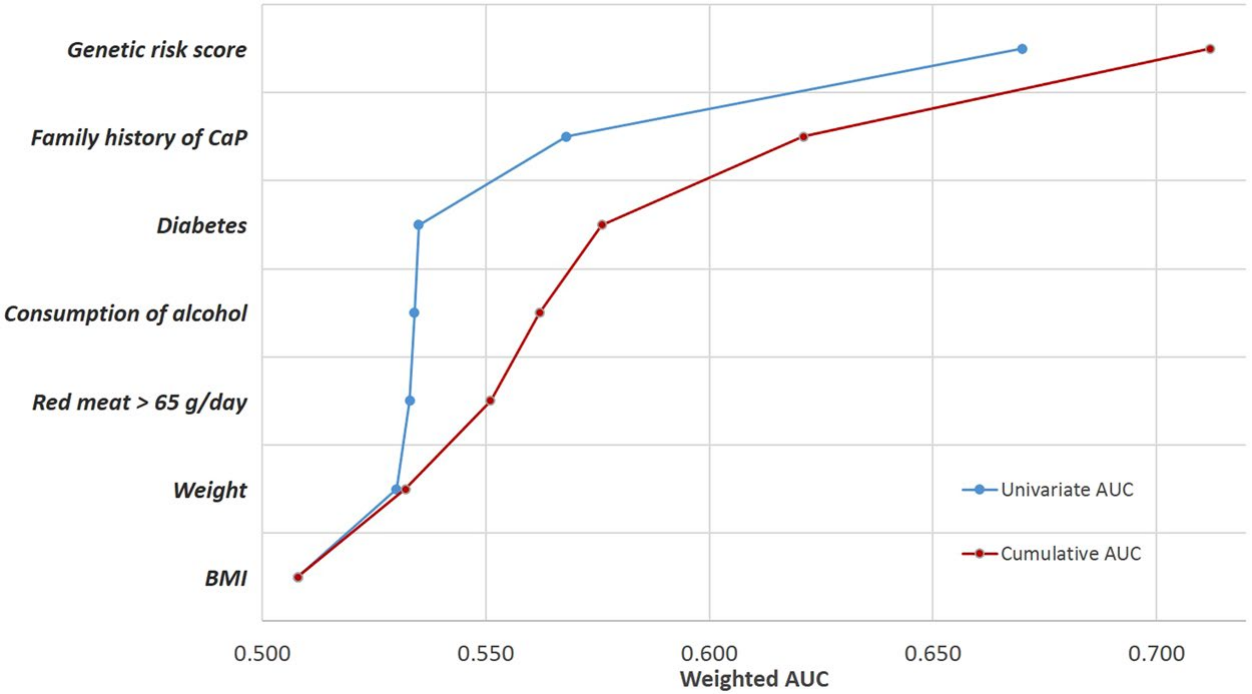
Individual and cumulative contribution of each factor to prostate cancer predictive accuracy. The area under the ROC curve (AUROC), as indicator of predictive accuracy for each variable in the risk model is shown. The left discontinuous (blue) line indicates the individual contribution of each variable, and the right continuous (red) line indicates the cumulative contribution, bottom to top. Environmental variables are sorted by increasing AUROC.

Figure 2 shows the contribution to PCa risk prediction that was estimated for modifiable environmental risk factors, family history and the genetic risk score. The individual (blue line) and cumulative (red line) represent the contribution of each environmental factor to the risk. The cumulative contribution of the five environmental factors resulted in an area under the roc curve (AUROC) of 0.57 (95% CI 0.54 to 0.60). Family history increased the AUROC to 0.62 (95% CI 0.59 to 0.64). The genetic risk score, on its own, had an AUROC of 0.66 (95% CI 0.63 to 0.68). When adding family history to the GRS model, the AUROC increased to 0.68 (95% CI 0.66 to 0.70); if the environmental score was also added, the AUROC reached 0.71 (95% CI 0.68 to 0.74).

The heterogeneity of prostate cancer plays an important and particular role with respect to the association with many of the environmental risk factors, although, when considering the heterogeneity of prostate cancers, we observed that the AUC was very similar in all strata observed. In Supplementary Table 3, we compared the AUC according to the age, the aggressiveness of the tumor (measured with the Gleason score) and the stage at diagnosis and we observed that, in general, the AUC was very similar in all the strata with respect to the global model, since the maximum observed change was in the 3rd decimal. The genetic score added the most to the predictive ability, reaching an AUROC of 0.75 in people under 65 years old (95% CI 0.71 to 0.79).

Improvement in risk prediction by adding more scores on GRS components was measured with three indicators: net reclassification index (NRI), integrated improvement of discrimination (IDI) and improvement in AUROC, the results of which are shown in Table 3. Adding ERS to GRS improved patient classification by 14% (NRI = 0.142) with marginal improvements in IDI and AUROC. The difference in the odds of having prostate cancer between cases and controls almost did not improve anything (IDI = 0.009). However, the addition of GRS to ERS scored 43% in NRI (NRI = 0.428) and the difference in odds of prostate cancer between cases and controls improved by 5% (IDI = 0.054).

**Table 3:** Improvement in risk prediction when adding more component scores.

Bootstrap results						
Base score	AUC (CI 95%)^*^	bias	Improvement in AUROC	p value**	Net Reclassification Improvement	Integrated Discrimination Improvement
GRS	0.630 (0.599–0.656)	0.0001				
GRS + ERS	0.645(0.618–0.676)	−0.0002	0.018	0.003	0.142	0.009
ERS	0.545 (0.513–574)	0.0010				
ERS + GRS	0.644 (0.616–0.667)	0.0008	0.09	<0.001	0.428	0.054

*AUC estimates bias-corrected confidence interval 95%.

**p value for the improvement in AUROC.

GRS: Genetic Risk Score.

ERS: Environmental Risk Score.

**Figure 3:**
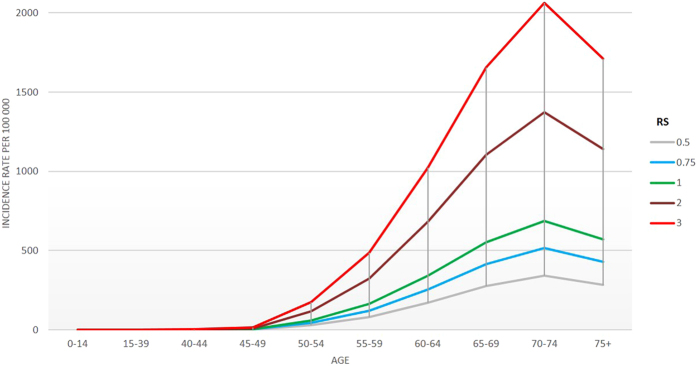
Estimation of prostate cancer incidence in Spain by age (years) and risk score. Color lines indicate age-specific incidence rates of prostate cancer per 100 000 individuals in Spain according to risk score (RS), for a selection of values. The incidence curve for the average individual corresponds to RS = 1. The risk score can be calculated as RS = 2.47^(ERS-0.94)^ * 3.32^FH^ * 2.05^(GRS-6.98)^, where ERS is the points in the environmental score (average 0.94 in the population), FH is the presence of family history of prostate cancer (0 = no, 1 = yes) and GRS is the points in the genetic score (average 6.98 in the population).

A simple calculation of the relative risk could be done through the following risk score (RS) equation: RS = 2.47^(ERS-0.94)^ * 3.32^FH^ * 2.05^(GRS-6.98)^. This is how Fig. 3 was constructed: An individual with the average exposure in the population (i.e.: with no family history (FH = 0), 0.94 points in the environmental score and 6.98 points in the genetic score) would have the average population risk (RS = 1). When applied to a specific subject, this equation gives his relative risk with respect to the average population; for instance, a subject with 1 points in the environmental score, family history and 7 points in the genetic score would have a RS of 3.5 times the average risk. These relative risk estimates can be applied to specific incidence rates for a population to derive the absolute risk estimates. For example, Spanish cancer incidence data was used to estimate the proportion of the population that might be included within a high-risk category sufficient to merit more intensive PCa screening and/or lifestyle intervention.

Figure 3 displays how age-specific incidence curves are shifted according to the risk score. It can be observed that, between 50 and 70 years of age, incidence grows exponentially when considering RS > 1. On the other hand, with RS < 1 incidence also grow, but in a smoother fashion. For instance, a hypothetical individual with an RS of 3 reaches the highest incidence level of the average population (RS = 1) between 55 and 64 years old, instead of between 70 and 74, as the latter does (10–20 years earlier, approximately).

## Discussion

We have evaluated the potential usefulness of a model for predicting risk of PCa, which combines modifiable risk factors (lifestyle) with family history of PCa and genetic risk score based on the susceptibility of 56 SNPs. We have observed that genetic susceptibility has a stronger predictive value than modifiable risk factors. While the added value of each SNP is small, the combination of 56 SNPs adds to the predictive ability of the risk model.

Although the identified SNPs have small effects (PCa risk only increases by 10% per allele overall), their addition seriously improves the prediction model. Thus, our analysis has shown that the genetic risk score has a much stronger predictive capacity than the score of environmental score (AUROC for the genetic score: 0.66 vs. AUROC from the environmental score: 0.57). This is consistent with the literature as the PCa hereditary component accounts for up to 42% of the risk^15, 16^, being the cancer with higher hereditary burden^15^. Moreover, twin studies have demonstrated 50% higher risk in monozygotic than dizygotic twins, which means that inherited factors are responsible for familial aggregation of PCa, well above that of environmental factors^15^. The inclusion of additional genetic variants of the established regions of susceptibility to prostate cancer improves the prediction of the disease. Our model improves the predictive accuracy reported by Agalliu using a genetic score for PCa with 31 SNPs^36^. However, Szulkin *et al*., using a polygenic risk score with 65 established susceptibility variants obtained a tenth more than we are reporting, AUROC = 0.67; moreover, when adding 68 new variants, Szultkin *et al*.’s genetic score only improved its performance until AUROC = 0.68^37^, which suggests that genetic scores elaborated by adding the main effects of a number of SNPs would be close to saturation. As the estimated inheritance for PCa is not fully explained with these scores, further research on gene-gene and gene-environment interactions is required, which could require original designs or huge amounts of data.

A possible application of our genetic score is its potential ability to classify people according to their genetic risk, which would be useful in developing risk-specific strategies for PCa screening. Although using prostate specific antigen (PSA) for PCa screening had been endorsed by several organizations^38–40^, the current USPSTF recommendation is against it^41^. The lack of well-established environmental risk factors makes it impossible to individualize recommendations for PSA screening according to individual risk. However, as genotyping techniques are getting cheaper, a genetic score could help in stratifying people according to their PCa genetic risk, making screening individualization doable.

On the other hand, our study confirms that family history of prostate cancer is the most important risk factor for PCa. Our relative risk estimate (OR = 3.32) is consistent with the relative risks among 2 and 4, which have been reported in other studies, including multiple reviews and meta-analyses^42, 43^.

Finally, we were not able to replicate the risk associated with low consumption of vegetables, fruits or fish, high alcohol consumption or spirits, milk, dairy foods, dietary calcium or vitamin D, smoking, obesity and lack of physical activity in leisure time. Only high intake of red meat was associated as an independent risk predictor of prostate cancer. On average, high consumption of red meat increases the risk of PCa by 31%. Similar results have been published relating the high consumption of meat and meat products with PCa risk^9, 44, 45^. Also, an alcohol consumption, between 8 and 21 grams day (second tertile) increased the risk of prostate cancer by 39%. Inconsistent results have been found on the relationship between alcohol consumption and prostate cancer. In a recent meta-analysis by Zhao J. *et al*.^46^ a significant dose-response relationship between the level of alcohol consumption and the risk of prostate cancer from low volume consumption (>1.3, <24 g per day) is shown for the first time. This is in line with our results. However, diabetes was associated with an independent protective predictor of prostate cancer (OR = 0.58, 95% CI 0.43 to 0.79). In our study, diabetes was associated with lower PCa risk only in those people who either did not take any treatment or had oral antidiabetic treatment. Diabetics treated with insulin did not reach statistical significance. Type 2 diabetes mellitus (T2DM) has consistently been associated with decreased risk of prostate cancer^47^. It was suggested that the decrease in risk is related to the use of anti‐diabetic drugs^48^. The most commonly prescribed anti‐diabetic drugs, the metformin has been recently investigated with inconsistent results; some studies have found a decreased risk of prostate cancer among metformin users^49, 50^, while others have found no association^51, 52^.

This study has some limitations. Firstly, our model on environmental factors has been developed in a retrospective case-control study, and was based on self-reported data. Therefore, measurement error and recall bias may have led to an underestimation of its predictive accuracy. Secondly, some differences in characteristics among cases and controls (namely, age, socio-economic level, BMI) suggest a possible selection bias, which could have been introduced via differential sample fraction. In order to reduce it, we adjusted all our analyses for a propensity score including those differential variables. Thirdly, as the cases were matched by age with controls, age cannot be included in the risk model, which could be desirable in a model like this. Finally, our study requires validation, as we developed our scores in a case-control study, without testing them in an independent sample.

In conclusion, we have evaluated the accuracy of a PCa predictive model that could be useful to stratify the population into risk categories and detection of PCa. In our model, genetic factors contribute more to PCa risk than modifiable risk factors, although further studies are needed to determine the generalizability, usefulness, and cost-effectiveness of the implementation of a genetic score as a pre-screening test for PCa. However, it should be noted that the cost of genotyping is decreasing, their determination has to be done only once in a life, and the data is likely to be useful for predicting the risk of different diseases not just cancer. Moreover, awareness of personal risk of PCa could trigger healthy changes in lifestyle of people at higher risk and therefore reduce the incidence of this tumor.

## Electronic supplementary material

Supplementary Information

## References

[1] Ferlay J (2015). Cancer incidence and mortality worldwide: sources, methods and major patterns in GLOBOCAN 2012. Int.J.Cancer.

[2] Bostwick DG (2004). Human prostate cancer risk factors. Cancer.

[3] Burks DA, Littleton RH (1992). The epidemiology of prostate cancer in black men. Henry Ford Hosp. Med. J..

[4] Crawford ED (2003). Epidemiology of prostate cancer. Urology.

[5] Hsing AW, Chokkalingam AP (2006). Prostate cancer epidemiology. Front. Biosci. J. Virtual Libr..

[6] Pienta KJ, Esper PS (1993). Risk factors for prostate cancer. Ann. Intern. Med..

[7] Dagnelie PC, Schuurman AG, Goldbohm RA, Van den Brandt PA (2004). Diet, anthropometric measures and prostate cancer risk: a review of prospective cohort and intervention studies. BJU Int..

[8] Kolonel LN, Altshuler D, Henderson BE (2004). The multiethnic cohort study: exploring genes, lifestyle and cancer risk. Nat. Rev. Cancer.

[9] Kolonel LNF (2001). meat, and prostate cancer. Epidemiol. Rev..

[10] Wolk A (2005). Diet, lifestyle and risk of prostate cancer. Acta Oncol. Stockh. Swed..

[11] Wilson KM, Giovannucci EL, Mucci LA (2012). Lifestyle and dietary factors in the prevention of lethal prostate cancer. Asian J. Androl..

[12] World Cancer Research Fund International/American Institute for Cancer Research Continuous Update Project Report. *Diet*, *Nutrition*, *Physical Activity*, *and Prostate Cancer* (2014).

[13] Markozannes G (2016). Diet, body size, physical activity and risk of prostate cancer: An umbrella review of the evidence. Eur. J. Cancer Oxf. Engl. 1990.

[14] Hjelmborg JB (2014). The heritability of prostate cancer in the Nordic Twin Study of Cancer. Cancer Epidemiol. Biomark. Prev. Publ. Am. Assoc. Cancer Res. Cosponsored Am. Soc. Prev. Oncol..

[15] Lichtenstein P (2000). Environmental and heritable factors in the causation of cancer–analyses of cohorts of twins from Sweden, Denmark, and Finland. N. Engl. J. Med..

[16] Schaid DJ (2004). The complex genetic epidemiology of prostate cancer. Hum. Mol. Genet..

[17] Seng KC, Seng CK (2008). The success of the genome-wide association approach: a brief story of a long struggle. Eur. J. Hum. Genet. EJHG.

[18] Eeles R (2014). The genetic epidemiology of prostate cancer and its clinical implications. Nat. Rev. Urol..

[19] Eeles, R. A. *et al*. Identification of 23 new prostate cancer susceptibility loci using the iCOGS custom genotyping array. *Nat*. *Genet*. **45**, 385–391, 391e1–2 (2013). 10.1038/ng.2560 PMC3832790 23535732

[20] Kote-Jarai Z (2008). Multiple novel prostate cancer predisposition loci confirmed by an international study: the PRACTICAL Consortium. Cancer Epidemiol. Biomark. Prev. Publ. Am. Assoc. Cancer Res. Cosponsored Am. Soc. Prev. Oncol..

[21] Castano-Vinyals, G. *et al*. Population-based multicase-control study in common tumors in Spain (MCC-Spain): rationale and study design. *Gac*.*Sanit*. doi:10.1016/j.gaceta.2014.12.003 (2015). 10.1016/j.gaceta.2014.12.003 25613680

[22] ciberesp.qxp − 03_Cuestionario-alimentario_09Nov09.pdf. Available at: http://www.mccspain.org/wp-content/uploads/2016/04/03_Cuestionario-alimentario_09Nov09.pdf (Accessed: 23rd June 2017).

[23] Cuzick J (2014). Prevention and Early Detection of Prostate Cancer. Lancet Oncol..

[24] Ainsworth BE (2000). Compendium of physical activities: an update of activity codes and MET intensities. Med. Sci. Sports Exerc..

[25] WHO | What is Moderate-intensity and Vigorous-intensity Physical Activity? *WHO* Available at: http://www.who.int/dietphysicalactivity/physical_activity_intensity/en/ (Accessed: 5th May 2017).

[26] Obesity: preventing and managing the global epidemic. Report of a WHO consultation. *World Health Organ*. *Tech*. *Rep*. *Ser*. **894**, i–xii, 1-253 (2000). 11234459

[27] Hindorff, L. *et al*. A Catalog of Published Genome-Wide Association Studies. www.genome.gov/gwastudies Available at: https://www.genome.gov/26525384/catalog-of-published-genomewide-association-studies/ (Accessed: 9th September 2016) (2015).

[28] Johnson AD (2008). SNAP: a web-based tool for identification and annotation of proxy SNPs using HapMap. Bioinformatics.

[29] SNAP P Search. Available at: http://archive.broadinstitute.org/mpg/snap/ldsearch.php (Accessed: 7th February 2017).

[30] Månsson R, Joffe MM, Sun W, Hennessy S (2007). On the estimation and use of propensity scores in case-control and case-cohort studies. Am. J. Epidemiol..

[31] Steyerberg, E. W. *Clinical Prediction Models*., doi:10.1007/978-0-387-77244-8 (Springer New York, 2009).

[32] Labarère J, Renaud B, Bertrand R, Fine MJ (2014). How to derive and validate clinical prediction models for use in intensive care medicine. Intensive Care Med..

[33] Pencina, M. J., D’Agostino, R. B., D’Agostino, R. B. & Vasan, R. S. Evaluating the added predictive ability of a new marker: from area under the ROC curve to reclassification and beyond. *Stat*. *Med*. **27**, 157–172; discussion 207–212 (2008). 10.1002/sim.2929 17569110

[34] Pencina MJ, D’Agostino RB, Steyerberg EW (2011). Extensions of net reclassification improvement calculations to measure usefulness of new biomarkers. Stat. Med..

[35] GLOBOCAN 2012 age-specific table. Available at: http://globocan.iarc.fr/old/age-specific_table_r.asp?selection=182724&title=Spain&sex=1&type=0&stat=0&window=1&sort=0&submit=%C2%A0Execute (Accessed: 12th September 2016).

[36] Agalliu I (2013). Characterization of SNPs associated with prostate cancer in men of Ashkenazic descent from the set of GWAS identified SNPs: impact of cancer family history and cumulative SNP risk prediction. PloS One.

[37] Szulkin R (2015). Prediction of individual genetic risk to prostate cancer using a polygenic score: Polygenic Prostate Cancer Risk Score. The Prostate.

[38] Smith RA (2011). Cancer screening in the United States, 2011: A review of current American Cancer Society guidelines and issues in cancer screening. CA. Cancer J. Clin..

[39] Loeb S (2014). Prostate cancer screening: highlights from the 29th European association of urology congress stockholm, sweden, april 11-15, 2014. Rev. Urol..

[40] Abrahamsson PA, Artibani W, Chapple CR, Wirth M (2010). [European Association of Urology. Position statement on screening for prostate cancer]. Actas Urol. Esp..

[41] Final Update Summary: Prostate Cancer: Screening - US Preventive Services Task Force. Available at: https://www.uspreventiveservicestaskforce.org/Page/Document/UpdateSummaryFinal/prostate-cancer-screening (Accessed: 17th October 2016) (2016).

[42] Johns LE, Houlston RS (2003). A systematic review and meta-analysis of familial prostate cancer risk. BJU Int..

[43] Bruner DW, Moore D, Parlanti A, Dorgan J, Engstrom P (2003). Relative risk of prostate cancer for men with affected relatives: systematic review and meta-analysis. Int. J. Cancer.

[44] Chan JM (1998). Dairy products, calcium, phosphorous, vitamin D, and risk of prostate cancer (Sweden). Cancer Causes Control CCC.

[45] Platz EA, Clinton SK, Giovannucci E (2008). Association between plasma cholesterol and prostate cancer in the PSA era. Int. J. Cancer.

[46] Zhao J, Stockwell T, Roemer A, Chikritzhs T (2016). Is alcohol consumption a risk factor for prostate cancer? A systematic review and meta-analysis. BMC Cancer.

[47] Tsilidis KK, Kasimis JC, Lopez DS, Ntzani EE, Ioannidis JPA (2015). Type 2 diabetes and cancer: umbrella review of meta-analyses of observational studies. BMJ.

[48] Murtola TJ, Tammela TLJ, Lahtela J, Auvinen A (2008). Antidiabetic medication and prostate cancer risk: a population-based case-control study. Am. J. Epidemiol..

[49] Preston MA (2014). Metformin use and prostate cancer risk. Eur. Urol..

[50] Tseng C-H (2014). Metformin significantly reduces incident prostate cancer risk in Taiwanese men with type 2 diabetes mellitus. Eur. J. Cancer Oxf. Engl. 1990.

[51] Tsilidis KK (2014). Metformin does not affect cancer risk: a cohort study in the U.K. Clinical Practice Research Datalink analyzed like an intention-to-treat trial. Diabetes Care.

[52] Häggström C (2017). Prospective study of Type 2 diabetes mellitus, anti‐diabetic drugs and risk of prostate cancer. Int. J. Cancer.

