# Supplementary material for: Risk Model for Prostate Cancer Using Environmental and Genetic Factors in the Spanish Multi-Case-Control (MCC) Study
Source: Sci Rep. 2017 Aug 21;7:8994. doi: 10.1038/s41598-017-09386-9 (PMC5566549; doi:10.1038/s41598-017-09386-9)

**TITLE PAGE**

**Full title:** Risk Model for Prostate Cancer Using Environmental and Genetic Factors in the Spanish Multi-Case-Control (MCC) Study

**Short title:** Risk Model for Prostate Cancer

**Authors:**

Inés Gómez-Acebo^1,2^, Trinidad Dierssen-Sotos^1,2^, Pablo Fernandez-Navarro^1,3^, Camilo Palazuelos^2^, Víctor Moreno^1,4^ Nuria Aragonés^1,3,5^,, Gemma Castaño-Vinyals^1,6,7,8^, Jose J. Jiménez-Monleón^1,9^, Jose Luis Ruiz-Cerdá^10^, Beatriz Pérez-Gómez^1,3,5^, José Manuel Ruiz-Dominguez^11^, Jessica Alonso Molero^2^, Marina Pollán^1,3^, Manolis Kogevinas^1,6,7,8^, Javier Llorca^1,2^

**Supplementary Table 1.** List of 56 SNPs selected and the genomic region and gene they belong to

| **Number** | **Genomic region** | **PCa risk** | **OR** | **PMID*** | **Proxy** |  | **risk allele** | **Known Gene** | **LD with** |
| --- | --- | --- | --- | --- | --- | --- | --- | --- | --- |
|  |  | **SNP** | **Published*** |  | **used** | **wild allele** |  |  |  |
| 1 | 1q21.3 | rs17599629 | 1.08 | 25217961 | rs267738 | T | T | CERS2 |  |
|  |  |  | 1.12 | 26034056 |  |  |  |  |  |
| 2 | 1q32.1 | rs4245739 | 1.10 | 23535732 |  | A | A | MDM4 |  |
|  |  |  | 1.14 | 23535733 |  |  |  |  |  |
|  |  |  | 1.19 | 24325915 |  |  |  |  |  |
|  |  |  | 1.09 | 27117709 |  |  |  |  |  |
| 3 | 2p11.2 | rs10187424 | 1.09 | 21743467 | rs1078004 | G | G | MAT2A |  |
|  |  |  | 1.11 | 26034056 |  |  |  |  |  |
| removed | 2p11.2 | rs3731827 | 1.13 | 26034056 | rs1561198 | C | C | VAMP8 | rs1078004 |
| 4 | 2p15 | rs6545977 |  | 19767753 |  | G | G | Unknown |  |
| 5 | 2p21 | rs1465618 | 1.08 | 19767753 |  | C | T | THADA |  |
| 6 | 2q31 | rs12621278 | 1.33 | 19767753 |  | A | G | ITGA6 |  |
| 7 | 2q37.3 | rs2292884 | 1.14 | 21743057 |  | A | G | MPLH |  |
| 8 | 3p12 | rs2660753 | 1.18 | 18264097 |  | C | T | Unknown |  |
|  |  |  | 1.19 | 26443449 |  |  |  |  |  |
| 9 | 3p12.1-2 | rs17181170 |  | 19767753 |  | G | G | LINC00506(no coding region) |  |
| 10 | 3p12.1-2 | rs7629490 | 1.06 | 21743057 |  | C | T | Unknown |  |
| 11 | 3q21 | rs10934853 | 1.12 | 19767754 |  | C | A | EEFSEC |  |
| 12 | 3q23 | rs6763931 | 7.40 | 18391951 | rs724016 | A | A | ZBTB38 |  |
|  |  |  | 0.07 | 19343178 |  |  |  |  |  |
|  |  |  | 1.04 | 21743467 |  |  |  |  |  |
|  |  |  | 0.02 | 25221879 |  |  |  |  |  |
|  |  |  | 0.05 | 25429064 |  |  |  |  |  |
| 13 | 4q22 | rs12500426 | 1.08 | 19767753 |  | C | C | PDLIM5 |  |
| 14 | 4q22 | rs17021918 | 1.11 | 19767753 |  | C | C | PDLIM5 |  |
| 15 | 5p12 | rs2121875 | 1.05 | 21743467 | rs1448044 | G | G | NA |  |
| 16 | 5q14.3 | rs4466137 |  | 17903305 |  | G | G | HAPLN1 |  |
| 17 | 6p12.2 | rs10498792 |  | 17903305 |  | T | T | PKHD1 |  |
| 18 | 6p21 | rs130067 | 1.05 | 21743467 |  | T | G | CCHCR1 |  |
| 19 | 6p21 | rs3096702 | 1.07 | 23535732 |  | G | A | NOTCH4 |  |
| 20 | 6p21.3 | rs115306967 | 1.06 | 25217961 | rs3129853 | G | G | NA |  |
| 21 | 6p22.1 | rs115457135 | 1.07 | 25217961 | rs3807031 | C | C | PPP1R11 |  |
| 22 | 6q22.2 | rs339331 | 1.22 | 20676098 |  | T | T | RFX6 |  |
|  |  |  | 1.12 | 26034056 |  |  |  |  |  |
|  |  |  | 1.28 | 26443449 |  |  |  |  |  |
| 23 | 6q25 | rs9364554 | 1.17 | 18264097 |  | C | T | SLC22A3 |  |
|  |  |  | 1.14 | 26034056 |  |  |  |  |  |
| 24 | 6q25.3 | rs7758229 | 1.28 | 21242260 |  | G | G | SLC22A3 |  |
|  |  |  | 1.15 | 25939597 |  |  |  |  |  |
| 25 | 7p12.3 | rs56232506 | 1.06 | 25217961 | rs11763044 | G | G | TNS3 |  |
| 26 | 7p15 | rs10486567 | 1.12 | 18264096 |  | G | G | JAZF1 |  |
|  |  |  | 1.17 | 26034056 |  |  |  |  |  |
| 27 | 7p15.3 | rs12155172 | 1.05 | 19767753 |  | G | G | Unknown |  |
|  |  |  | 1.11 | 23535732 |  |  |  |  |  |
| 28 | 7q21 | rs6465657 | 1.12 | 18264097 |  | T | C | LMTK2 |  |
| 29 | 8p21.2 | rs1512268 | 1.18 | 19767753 |  | C | T | Unknown |  |
|  |  |  | 1.35 | 22923026 |  |  |  |  |  |
|  |  |  | 1.17 | 26034056 |  |  |  |  |  |
|  |  |  | 1.32 | 26443449 |  |  |  |  |  |
| 30 | 8q24 | rs6983267 | 1.26 | 17401363 |  | G | G | CCAT2(no coding region) |  |
|  |  |  | 1.27 | 17618284 |  |  |  |  |  |
|  |  |  | 1.28 | 18264096 |  |  |  |  |  |
|  |  |  | 1.42 | 18264097 |  |  |  |  |  |
|  |  |  | 1.24 | 18372905 |  |  |  |  |  |
|  |  |  | 1.18 | 21242260 |  |  |  |  |  |
|  |  |  | 1.20 | 21743057 |  |  |  |  |  |
|  |  |  | 1.13 | 23266556 |  |  |  |  |  |
|  |  |  | 1.36 | 24740154 |  |  |  |  |  |
|  |  |  | 0.29 | 24753544 |  |  |  |  |  |
|  |  |  | 1.14 | 24836286 |  |  |  |  |  |
|  |  |  | 1.23 | 25939597 |  |  |  |  |  |
|  |  |  | 1.25 | 26034056 |  |  |  |  |  |
|  |  |  | 1.12 | 26151821 |  |  |  |  |  |
|  |  |  | 1.14 | 26965516 |  |  |  |  |  |
| 31 | 8q24.21 | rs13252298 | 1.12 | 21743057 |  | A | A | PRNCR1 |  |
|  |  |  | 1.11 | 26034056 |  |  |  |  |  |
| 32 | 8q24.21 | rs16902094 | 1.21 | 19767754 |  | A | G | CASC8(no coding region) |  |
| 33 | 8q24.21 | rs16901979 | 1.79 | 17401366 |  | C | A | MYC |  |
|  |  |  | 1.80 | 19767754 |  |  |  |  |  |
|  |  |  | 1.65 | 25939597 |  |  |  |  |  |
|  |  |  | 1.39 | 26443449 |  |  |  |  |  |
| 34 | 8q24.21 | rs445114 | 1.14 | 19767754 |  | T | T | CASC8(no coding region) |  |
|  |  |  | 1.22 | 21743057 |  |  |  |  |  |
| 35 | 8q24.21 | rs4242382 | 1.66 | 18264096 |  | G | A | Unknown |  |
|  |  |  | 1.45 | 25939597 |  |  |  |  |  |
| 36 | 8q24.21 | rs1016343 | 1.37 | 18264097 |  | C | T | PRNCR1 |  |
|  |  |  | 1.31 | 21743057 |  |  |  |  |  |
|  |  |  | 1.25 | 26034056 |  |  |  |  |  |
| removed | 8q24.21 | rs1447295 | 1.43 | 17401363 |  | C | A | CASC8(no coding region) | rs4242382, rs4242384 |
|  |  |  | 1.60 | 17401366 |  |  |  |  |  |
|  |  |  | 1.58 | 19767754 |  |  |  |  |  |
|  |  |  | 0.51 | 24753544 |  |  |  |  |  |
|  |  |  | 1.54 | 26443449 |  |  |  |  |  |
| removed | 8q24.21 | rs4242384 | 1.88 | 18264097 |  | A | C | Unknown | rs4242382, rs1447295 |
|  |  |  | 1.56 | 21743057 |  |  |  |  |  |
| 37 | 10q11.23 | rs10993994 | 1.16 | 18264096 |  | C | T | MSMB |  |
|  |  |  | 1.25 | 18264097 |  |  |  |  |  |
|  |  |  | 9.20 | 21160077 |  |  |  |  |  |
|  |  |  | 1.18 | 21743057 |  |  |  |  |  |
|  |  |  | 0.12 | 23269536 |  |  |  |  |  |
|  |  |  | 1.25 | 23555189 |  |  |  |  |  |
|  |  |  | 1.32 | 24740154 |  |  |  |  |  |
|  |  |  | 0.40 | 24753544 |  |  |  |  |  |
|  |  |  | 1.16 | 25939597 |  |  |  |  |  |
|  |  |  | 1.21 | 26034056 |  |  |  |  |  |
|  |  |  | 1.20 | 26443449 |  |  |  |  |  |
| 38 | 10q11.23 | rs3123078 |  | 19767753 |  | T | C | Unknown |  |
| 39 | 10q26.13 | rs4962416 | 1.17 | 18264096 |  | T | C | CTBP2 |  |
| removed | 11q13 | rs7931342 | 1.19 | 18264097 |  | G | T | Unknown | rs10896449 |
|  |  |  | 1.30 | 24740154 |  |  |  |  |  |
| 40 | 11q13.2 | rs10896449 | 1.10 | 18264096 |  | G | G | Unknown |  |
|  |  |  | 1.20 | 26034056 |  |  |  |  |  |
| 41 | 11q13.2 | rs11228565 | 1.23 | 19767754 |  | G | A | Unknown |  |
| 42 | 11q13.2 | rs7130881 | 1.31 | 21743057 |  | A | G | Unknown |  |
| 43 | 12q13.2 | rs902774 | 1.17 | 21743057 |  | G | A | Unknown |  |
|  |  |  | 1.16 | 26034056 |  |  |  |  |  |
| 44 | 13q33.2 | rs1529276 |  | 17903305 |  | T | T | Unknown |  |
| 45 | 17q12 | rs4430796 | 1.22 | 17603485 |  | G | A | HNF1B |  |
|  |  |  | 1.18 | 18264096 |  |  |  |  |  |
|  |  |  | 1.19 | 19767754 |  |  |  |  |  |
|  |  |  | 1.14 | 20581827 |  |  |  |  |  |
|  |  |  | 9.40 | 21160077 |  |  |  |  |  |
|  |  |  | 1.19 | 21499250 |  |  |  |  |  |
|  |  |  | 1.19 | 22961080 |  |  |  |  |  |
|  |  |  | 1.11 | 23945395 |  |  |  |  |  |
|  |  |  | 1.13 | 24509480 |  |  |  |  |  |
|  |  |  | 1.23 | 26443449 |  |  |  |  |  |
| 46 | 17q21.2 | rs7501939 | 1.41 | 18264097 |  | C | C | HNF1B |  |
|  |  |  | 1.19 | 21743057 |  |  |  |  |  |
|  |  |  | 1.28 | 25877299 |  |  |  |  |  |
|  |  |  | 1.17 | 26034056 |  |  |  |  |  |
| 47 | 17q24 | rs1859962 | 1.20 | 17603485 |  | T | G | CASC17 |  |
|  |  |  | 1.26 | 18264097 |  |  |  |  |  |
|  |  |  | 1.27 | 21743057 |  |  |  |  |  |
|  |  |  | 1.13 | 26034056 |  |  |  |  |  |
| 48 | 19q13 | rs8102476 | 1.12 | 19767754 |  | C | T | Unknown |  |
| 49 | 19q13 | rs17632542 | 39.10 | 21160077 |  | T | T | KLK3 |  |
|  |  |  | 1.85 | 24740154 |  |  |  |  |  |
|  |  |  | 0.73 | 24753544 |  |  |  |  |  |
| 50 | 19q13 | rs2735839 | 1.20 | 18264097 |  | G | G | Unknown |  |
|  |  |  | 0.19 | 23269536 |  |  |  |  |  |
|  |  |  | 1.17 | 26034056 |  |  |  |  |  |
| 51 | 19q13.33 | rs2659124 | 1.18 | 26034056 | rs174776 | C | C | KLK3 |  |
| 52 | 20q13.33 | rs6062509 | 1.12 | 23535732 | rs4809330 | G | G | ZGPAT |  |
| 53 | 22q13 | rs5759167 | 1.16 | 19767753 |  | G | G | Unknown |  |
|  |  |  | 1.18 | 25939597 |  |  |  |  |  |
|  |  |  | 1.13 | 26034056 |  |  |  |  |  |
| removed | 22q13.1 | rs11704416 | 1.05 | 23065704 | rs2018458 | C | C | TNRC6B | rs9623117 |
| 54 | 22q13.1 | rs9623117 | 1.18 | 19117981 |  | T | C | TNRC6B |  |
| 55 | 22q13.2 | rs742134 | 1.16 | 21743057 |  | G | G | BIK |  |
| removed | Xp11.22 | rs1327301 |  | 19767753 |  | C | T | Unknown | rs5945572 |
| 56 | Xp11.22 | rs5945572 | 1.23 | 18264098 |  | G | A | Unknown |  |

LD: linkage disequilibrium. All SNP with LD: SNP >0.8 were removed. OR: Odds Ratio. PMID: PubMed ID of the article the OR was published.

OR Published and PMID Obtained from the GWAS Catalog*

Wild allele defined as the most frequent allele in our data. Risk allele define as the allele with higher prostate cancer risk in our data

| **Number** | **SNP** | **genotype** | **Controls** | **Cases** | **0R(95% CI)** | **P** |  | **Number** | **SNP** | **genotype** | **Controls** | **Cases** | **0R(95% CI)** | **P** |
| --- | --- | --- | --- | --- | --- | --- | --- | --- | --- | --- | --- | --- | --- | --- |
| **1** | rs267738 | TT | 662 | 549 | 1 ( 1 - 1 ) | . |  | **16** | rs4466137 | GG | 618 | 504 | 1 ( 1 - 1 ) | . |
|  |  | gT | 304 | 243 | 0.96 (0.78-1.17) | 0.668 |  |  |  | tG | 353 | 273 | 0.94 ( 0.77 - 1.15 ) | 0.536 |
|  |  | gg | 40 | 26 | 0.82 (0.49-1.36) | 0.435 |  |  |  | tt | 35 | 41 | 1.31 ( 0.81 - 2.1 ) | 0.271 |
| **2** | rs4245739 | AA | 459 | 388 | 1 ( 1 - 1 ) | . |  | **17** | rs10498792 | TT | 829 | 691 | 1 ( 1 - 1 ) | . |
|  |  | cA | 454 | 349 | 0.87 ( 0.72 - 1.07 ) | 0.186 |  |  |  | cT | 166 | 115 | 0.87 ( 0.67 - 1.13 ) | 0.296 |
|  |  | cc | 93 | 81 | 1 ( 0.71 - 1.39 ) | 0.979 |  |  |  | cc | 8 | 9 | 1.36 ( 0.51 - 3.6 ) | 0.541 |
| **3** | rs1078004 | GG | 245 | 195 | 1 ( 1 - 1 ) | . |  | **18** | rs130067 | TT | 671 | 524 | 1 ( 1 - 1 ) | . |
|  |  | cG | 509 | 421 | 1.02 (0.81-1.28) | 0.898 |  |  |  | gT | 297 | 265 | 1.13 ( 0.92 - 1.38 ) | 0.257 |
|  |  | cc | 252 | 202 | 1.02 (0.78-1.34) | 0.865 |  |  |  | gg | 38 | 29 | 1.07 ( 0.64 - 1.77 ) | 0.805 |
| **4** | rs6545977 | GG | 269 | 266 | 1 ( 1 - 1 ) | . |  | **19** | rs3096702 | GG | 574 | 447 | 1 ( 1 - 1 ) | . |
|  |  | aG | 498 | 391 | 0.8 ( 0.65 - 1 ) | 0.054 |  |  |  | aG | 360 | 320 | 1.14 ( 0.94 - 1.4 ) | 0.185 |
|  |  | aa | 239 | 161 | **0.72 ( 0.55 - 0.94 )** | **0.017** |  |  |  | aa | 72 | 51 | 0.87 ( 0.59 - 1.28 ) | 0.473 |
| **5** | rs1465618 | CC | 654 | 503 | 1 ( 1 - 1 ) | . |  | **20** | rs3129853 | GG | 609 | 497 | 1 ( 1 - 1 ) | . |
|  |  | tC | 304 | 268 | 1.14 ( 0.93 - 1.4 ) | 0.205 |  |  |  | aG | 349 | 273 | 0.95 (0.78-1.16) | 0.613 |
|  |  | tt | 48 | 47 | 1.32 ( 0.86 - 2.03 ) | 0.214 |  |  |  | Aa | 46 | 47 | 1.25 (0.82-1.92) | 0.299 |
| **6** | rs12621278 | AA | 941 | 755 | 1 ( 1 - 1 ) | . |  | **21** | rs3807031 | CC | 746 | 586 | 1 ( 1 - 1 ) | . |
|  |  | gA | 65 | 61 | 1.27 ( 0.88 - 1.85 ) | 0.205 |  |  |  | aC | 236 | 209 | 1.10 (0.89-1.37) | 0.387 |
|  |  | gg | 0 | 2 | 1 ( 1 - 1 ) | . |  |  |  | aa | 24 | 23 | 1.16 (0.65-2.08) | 0.623 |
| **7** | rs2292884 | AA | 614 | 482 | 1 ( 1 - 1 ) | . |  | **22** | rs339331 | TT | 573 | 483 | 1 ( 1 - 1 ) | . |
|  |  | gA | 328 | 290 | 1.1 ( 0.9 - 1.35 ) | 0.356 |  |  |  | cT | 377 | 280 | 0.87 ( 0.71 - 1.06 ) | 0.168 |
|  |  | gg | 64 | 46 | 0.88 ( 0.59 - 1.33 ) | 0.554 |  |  |  | cc | 54 | 39 | 0.85 ( 0.55 - 1.32 ) | 0.464 |
| **8** | rs2660753 | CC | 739 | 554 | 1 ( 1 - 1 ) | . |  | **23** | rs9364554 | CC | 586 | 434 | 1 ( 1 - 1 ) | . |
|  |  | tC | 248 | 240 | **1.34 ( 1.08 - 1.66 )** | **0.008** |  |  |  | tC | 364 | 332 | **1.23 ( 1.01 - 1.50 )** | **0.032** |
|  |  | tt | 19 | 24 | 1.76 ( 0.94 - 3.31 ) | 0.078 |  |  |  | tt | 56 | 52 | 1.24 ( 0.82 - 1.86 ) | 0.303 |
| **9** | rs17181170 | GG | 256 | 224 | 1 ( 1 - 1 ) | . |  | **24** | rs7758229 | GG | 516 | 383 | 1 ( 1 - 1 ) | . |
|  |  | aG | 501 | 438 | 0.95 ( 0.76 - 1.20 ) | 0.857 |  |  |  | tG | 400 | 365 | **1.23 (1.01-1.50)** | **0.035** |
|  |  | aa | 249 | 151 | **0.67 ( 0.51 - 0.88 )** | **0.004** |  |  |  | tt | 90 | 70 | 1.03 (0.73-1.46) | 0.849 |
| **10** | rs7629490 | CC | 467 | 331 | 1 ( 1 - 1 ) | . |  | **25** | rs11763044 | GG | 288 | 206 | 1 ( 1 - 1 ) | . |
|  |  | tC | 445 | 399 | **1.24 ( 1.02 - 1.52 )** | **0.031** |  |  |  | aG | 501 | 435 | 1.21 (0.97-1.51) | 0.089 |
|  |  | tt | 94 | 88 | 1.35 ( 0.97 - 1.88 ) | 0.074 |  |  |  | aa | 217 | 174 | 1.11 (0.85-1.45) | 0.453 |
| **11** | rs10934853 | CC | 544 | 408 | 1 ( 1 - 1 ) | . |  | **26** | rs10486567 | GG | 592 | 536 | 1 ( 1 - 1 ) | . |
|  |  | aC | 389 | 326 | 1.15 ( 0.94 - 1.4 ) | 0.177 |  |  |  | aG | 352 | 255 | **0.77 ( 0.63 - 0.94 )** | **0.010** |
|  |  | aa | 73 | 84 | **1.56 ( 1.1 - 2.2 )** | **0.012** |  |  |  | aa | 61 | 27 | **0.5 ( 0.31 - 0.8 )** | **0.004** |
| **12** | rs724016 | AA | 416 | 351 | 1 ( 1 - 1 ) | . |  | **27** | rs12155172 | GG | 647 | 533 | 1 ( 1 - 1 ) | . |
|  |  | gA | 457 | 357 | 0.91 (0.75-1.11) | 0.360 |  |  |  | aG | 317 | 252 | 0.94 ( 0.77 - 1.16 ) | 0.587 |
|  |  | gg | 133 | 110 | 0.99 (0.74-1.32) | 0.936 |  |  |  | aa | 42 | 33 | 0.94 ( 0.58 - 1.52 ) | 0.804 |
| **13** | rs12500426 | CC | 284 | 235 | 1 ( 1 - 1 ) | . |  | **28** | rs6465657 | TT | 348 | 238 | 1 ( 1 - 1 ) | . |
|  |  | aC | **496** | **402** | 1 ( 0.8 - 1.25 ) | 0.989 |  |  |  | cT | 466 | 427 | **1.34 ( 1.08 - 1.67 )** | **0.007** |
|  |  | aa | 226 | 180 | 0.97 ( 0.74 - 1.26 ) | 0.802 |  |  |  | cc | 192 | 153 | 1.17 ( 0.89 - 1.54 ) | 0.261 |
| **14** | rs17021918 | CC | 413 | 365 | 1 ( 1 - 1 ) | . |  | **29** | rs1512268 | CC | 254 | 178 | 1 ( 1 - 1 ) | . |
|  |  | tC | 474 | 363 | 0.89 ( 0.73 - 1.08 ) | 0.237 |  |  |  | tC | 497 | 419 | 1.2 ( 0.95 - 1.52 ) | 0.135 |
|  |  | tt | 118 | 86 | 0.89 ( 0.65 - 1.23 ) | 0.482 |  |  |  | tt | 255 | 221 | 1.21 ( 0.92 - 1.58 ) | 0.173 |
| **15** | rs1448044 | GG | 406 | 289 | 1 ( 1 - 1 ) | . |  | **30** | rs6983267 | GG | 330 | 282 | 1 ( 1 - 1 ) | . |
|  |  | aG | 486 | 392 | 1.15 (0.94-1.41) | 0.187 |  |  |  | tG | 485 | 399 | 0.94 ( 0.76 - 1.17 ) | 0.592 |
|  |  | aa | 97 | 97 | **1.41 (1.02-1.95)** | **0.036** |  |  |  | tt | 191 | 137 | 0.81 ( 0.61 - 1.06 ) | 0.128 |

**Supplementary Table 2. Relationship between 56 single nucleotide polymorphisms and prostate cancer in MCC-Spain. Reference category: homozygous to most frequent allele**

**Table supplementary 2. (continued) Relationship between 56 single nucleotide polymorphisms and prostate cancer in MCC-Spain. Reference category: homozygous to most frequent allele**

| **Number** | **SNP** | **genotype** | **Controls** | **Cases** | **0R(95% CI)** | **P** |  | **Number** | **SNP** | **genotype** | **Controls** | **Cases** | **0R(95% CI)** | **P** |
| --- | --- | --- | --- | --- | --- | --- | --- | --- | --- | --- | --- | --- | --- | --- |
| **31** | rs13252298 | AA | 529 | 453 | 1 ( 1 - 1 ) | . |  | **44** | rs1529276 | TT | 577 | 482 | 1 ( 1 - 1 ) | . |
|  |  | gA | 393 | 312 | 0.92 ( 0.75 - 1.12 ) | 0.418 |  |  |  | aT | 367 | 291 | 0.96 ( 0.78 - 1.17 ) | 0.656 |
|  |  | gg | 84 | 53 | 0.75 ( 0.52 - 1.1 ) | 0.141 |  |  |  | aa | 62 | 45 | 0.92 ( 0.61 - 1.39 ) | 0.685 |
| **32** | rs16902094 | AA | 766 | 606 | 1 ( 1 - 1 ) | . |  | **45** | rs4430796 | GG | 299 | 205 | 1 ( 1 - 1 ) | . |
|  |  | gA | 229 | 194 | 1.1 ( 0.88 - 1.38 ) | 0.396 |  |  |  | aG | 467 | 408 | **1.32 ( 1.06 - 1.66 )** | **0.015** |
|  |  | gg | 11 | 18 | 2.06 ( 0.94 - 4.50 ) | 0.069 |  |  |  | aa | 240 | 203 | 1.28 ( 0.98 - 1.66 ) | 0.070 |
| **33** | rs16901979 | CC | 936 | 750 | 1 ( 1 - 1 ) | . |  | **46** | rs7501939 | CC | 350 | 301 | 1 ( 1 - 1 ) | . |
|  |  | aC | 69 | 66 | 1.16 (0.81-1.65) | 0.415 |  |  |  | tC | 473 | 396 | 0.97 ( 0.79 - 1.2 ) | 0.785 |
|  |  | aa | 1 | 2 | 2.31 (0.21-25.61) | 0.495 |  |  |  | tt | 183 | 121 | **0.75 ( 0.56 - 0.99 )** | **0.042** |
| **34** | rs445114 | TT | 367 | 356 | 1 ( 1 - 1 ) | . |  | **47** | rs1859962 | TT | 263 | 198 | 1 ( 1 - 1 ) | . |
|  |  | cT | 482 | 342 | **0.73 ( 0.6 - 0.9 )** | **0.003** |  |  |  | gT | 483 | 381 | 1.08 ( 0.86 - 1.36 ) | 0.510 |
|  |  | cc | 152 | 98 | **0.64 ( 0.47 - 0.86 )** | **0.004** |  |  |  | gg | 243 | 225 | 1.23 ( 0.95 - 1.6 ) | 0.121 |
| **35** | rs4242382 | GG | 851 | 662 | 1 ( 1 - 1 ) | . |  | **48** | rs8102476 | CC | 405 | 310 | 1 ( 1 - 1 ) | . |
|  |  | aG | 146 | 149 | **1.3 ( 1 - 1.68 )** | **0.047** |  |  |  | tC | 457 | 379 | 1.09 ( 0.88 - 1.33 ) | 0.437 |
|  |  | aa | 9 | 7 | 1.05 ( 0.38 - 2.88 ) | 0.969 |  |  |  | tt | 144 | 129 | 1.18 ( 0.88 - 1.56 ) | 0.268 |
| **36** | rs1016343 | CC | 603 | 463 | 1 ( 1 - 1 ) | . |  | **49** | rs17632542 | TT | 879 | 746 | 1 ( 1 - 1 ) | . |
|  |  | tC | 360 | 297 | 1.05 ( 0.86 - 1.28 ) | 0.629 |  |  |  | cT | 126 | 71 | **0.66 ( 0.48 - 0.9 )** | **0.008** |
|  |  | tt | 43 | 58 | **1.71 ( 1.12 - 2.62 )** | **0.013** |  |  |  | cc | 1 | 1 | 2.09 ( 0.12 - 35.57 ) | 0.609 |
| **37** | rs10993994 | CC | 326 | 225 | 1 ( 1 - 1 ) | . |  | **50** | rs2735839 | GG | 732 | 633 | 1 ( 1 - 1 ) | . |
|  |  | tC | 508 | 401 | 1.15 ( 0.92 - 1.43 ) | 0.208 |  |  |  | aG | 249 | 175 | 0.83 ( 0.66 - 1.03 ) | 0.095 |
|  |  | tt | 172 | 192 | **1.64 ( 1.25 - 2.16 )** | **0.000** |  |  |  | aa | 25 | 10 | **0.46 ( 0.22 - 0.98 )** | **0.043** |
| **38** | rs3123078 | TT | 289 | 194 | 1 ( 1 - 1 ) | . |  | **51** | rs174776 | CC | 768 | 646 | 1 ( 1 - 1 ) | . |
|  |  | cT | 513 | 396 | 1.17 ( 0.93 - 1.47 ) | 0.180 |  |  |  | tC | 222 | 160 | 0.87 (0.69-1.09) | 0.219 |
|  |  | cc | 204 | 228 | **1.71 ( 1.31 - 2.24 )** | **0.000** |  |  |  | tt | 16 | 11 | 0.85 (0.39-1.85) | 0.681 |
| **39** | rs4962416 | TT | 499 | 377 | 1 ( 1 - 1 ) | . |  | **52** | rs4809330 | GG | 524 | 449 | 1 ( 1 - 1 ) | . |
|  |  | cT | 411 | 362 | 1.17 ( 0.96 - 1.43 ) | 0.112 |  |  |  | aG | 413 | 309 | 0.87 (0.72-1.06) | 0.165 |
|  |  | cc | 96 | 79 | 1.11 ( 0.79 - 1.55 ) | 0.554 |  |  |  | aa | 69 | 60 | 1.05 (0.73-1.53) | 0.790 |
| **40** | rs10896449 | GG | 273 | 283 | 1 ( 1 - 1 ) | . |  | **53** | rs5759167 | GG | 248 | 245 | 1 ( 1 - 1 ) | . |
|  |  | aG | 525 | 397 | **0.74 ( 0.6 - 0.92 )** | **0.006** |  |  |  | tG | 511 | 405 | 0.81 ( 0.65 - 1.02 ) | 0.072 |
|  |  | aa | 208 | 138 | **0.64 ( 0.49 - 0.85 )** | **0.002** |  |  |  | tt | 247 | 168 | **0.7 ( 0.53 - 0.91 )** | **0.008** |
| **41** | rs11228565 | GG | 601 | 428 | 1 ( 1 - 1 ) | . |  | **54** | rs9623117 | TT | 610 | 468 | 1 ( 1 - 1 ) | . |
|  |  | aG | 367 | 330 | **1.26 ( 1.03 - 1.54 )** | **0.022** |  |  |  | cT | 366 | 301 | 1.11 ( 0.91 - 1.36 ) | 0.288 |
|  |  | aa | 38 | 60 | **2.28 ( 1.48 - 3.52 )** | **0.000** |  |  |  | cc | 30 | 49 | **2.16 ( 1.34 - 3.5 )** | **0.002** |
| **42** | rs7130881 | AA | 660 | 476 | 1 ( 1 - 1 ) | . |  | **55** | rs742134 | GG | 701 | 613 | 1 ( 1 - 1 ) | . |
|  |  | gA | 316 | 299 | **1.32 ( 1.08 - 1.62 )** | **0.007** |  |  |  | aG | 275 | 191 | **0.80( 0.64 - 0.99 )** | **0.044** |
|  |  | gg | 30 | 43 | **2.12 ( 1.3 - 3.47 )** | **0.003** |  |  |  | aa | 30 | 14 | **0.52 ( 0.27 - 1.00)** | **0.049** |
| **43** | rs902774 | GG | 781 | 602 | 1 ( 1 - 1 ) | . |  | **56** | rs5945572 | GG | 648 | 484 | 1 ( 1 - 1 ) | . |
|  |  | aG | 211 | 201 | 1.21 ( 0.96 - 1.51 ) | 0.100 |  |  |  |  |  |  | ( - ) |  |
|  |  | aa | 14 | 14 | 1.25 ( 0.58 - 2.68 ) | 0.571 |  |  |  | aa | 358 | 334 | **1.29 ( 1.07 - 1.57 )** | **0.009** |

**Supplementary table 3. Area under the roc curve between environmental risk score, family history, genetic risk score and the risk of prostate cancer according to characteristics of tumor (Gleason Score).**

|  | | | **AUC (95% CI)** | | |
| --- | --- | --- | --- | --- | --- |
|  |  |  | **ESR** | **ESR+FH** | **ESR+FH+GRS** |
| **ALL** | | | 0.57(0.54-0.60) | 0.62(0.59-0.64) | 0.71(0.68-0.74) |
| **GLEASON SCORE** | **Low risk ≤6** | **N=368** | 0.60(0.56-0.63) | 0.64(0.60-0.67) | 0.71(0.68-0.75) |
|  | **Intermediate =7** | **N=320** | 0.56(0.52-0.60) | 0.62(0.58-0.66) | 0.74(0.70-0.77) |
|  | **High risk >7** | **N=113** | 0.58(0.51-0.64) | 0.60(0.54-0.66) | 0.69(0.62-0.75) |
| **STAGE 1** | **t2a or t2b** | **N=629*** | 0.57(0.54-0.60) | 0.62(0.59-0.65) | 0.72(0.69-0.74) |
|  | **t2c** | **N=122*** | 0.59(0.54-0.65) | 0.63(0.57-0.69) | 0.70(0.65-0.76) |
| **AGES** | **<65 years** | **N=410** | 0.59(0.54-0.63) | 0.65(0.60-0.69) | 0.75(0.71-0.79) |
|  | **≥65 years** | **N=596** | 0.60(0.56-0.63) | 0.62(0.58-0.66) | 0.71(0.67-0.74) |

Stage 1 cancer is found only in the part of the body where it started it is localized

*Excluded the stage regional (spread to regional lymph nodes) and distant (cancer has metastasized.

AUC: Area under the Roc Curve

ERS: Environmental Risk Score

FH: family history

GRS: Genetic Risk Score

**Figure Legends**

**Supplementary Figure 1.**

**Distribution and PCa risk of the environmental risk score in cases and controls**

The left axis scale indicates the OR for PCa according to deciles of points in the environmental score. The decile 1 (0.23-0.61 points) has been selected as reference category (OR=1). The right axis scale indicates the proportion of cases and controls shown in bars for each decile of environmental score. Parentheses include the points in the score.


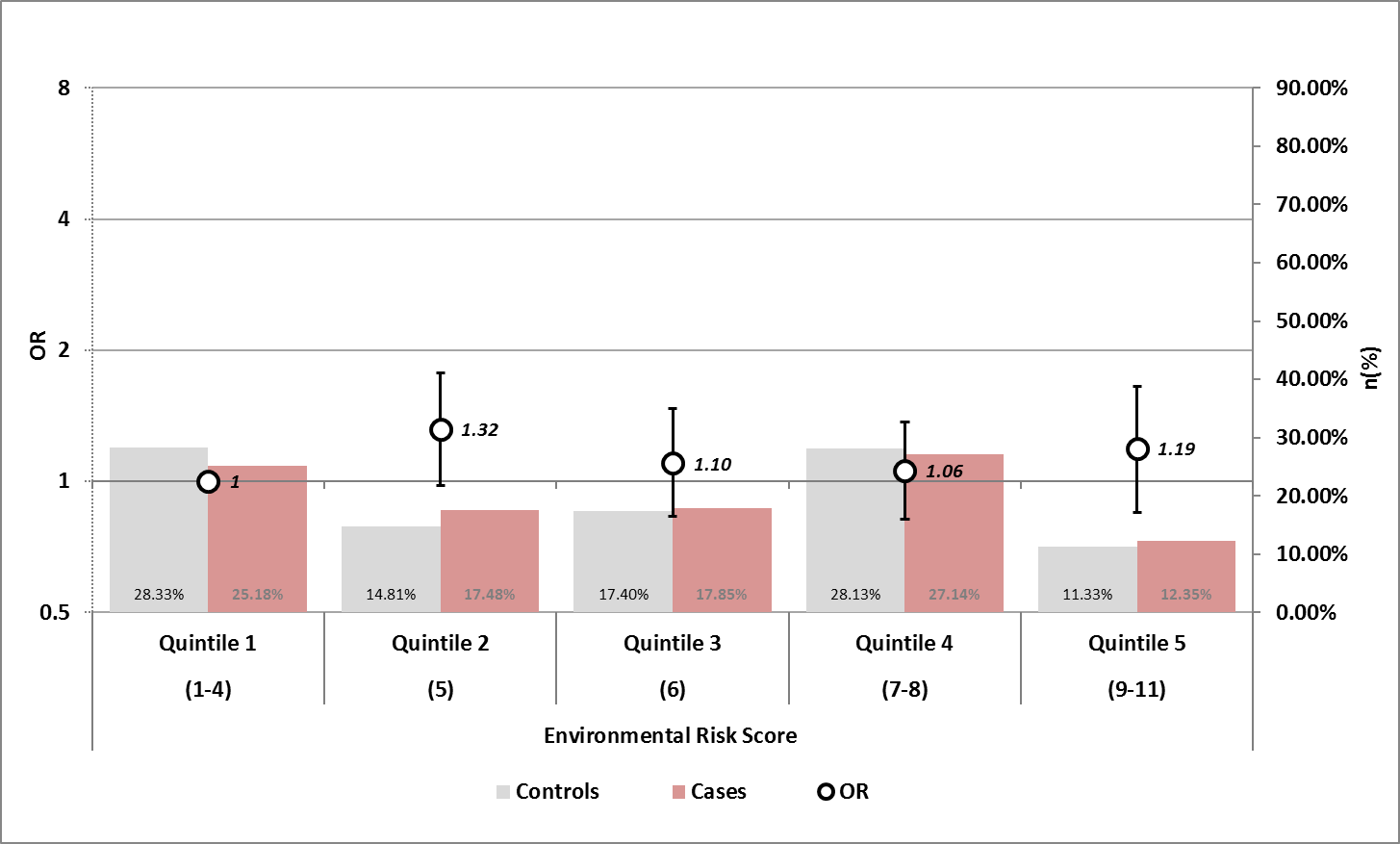


**Supplementary Figure 1.**

**Distribution and PCa risk of the environmental risk score in cases and controls**


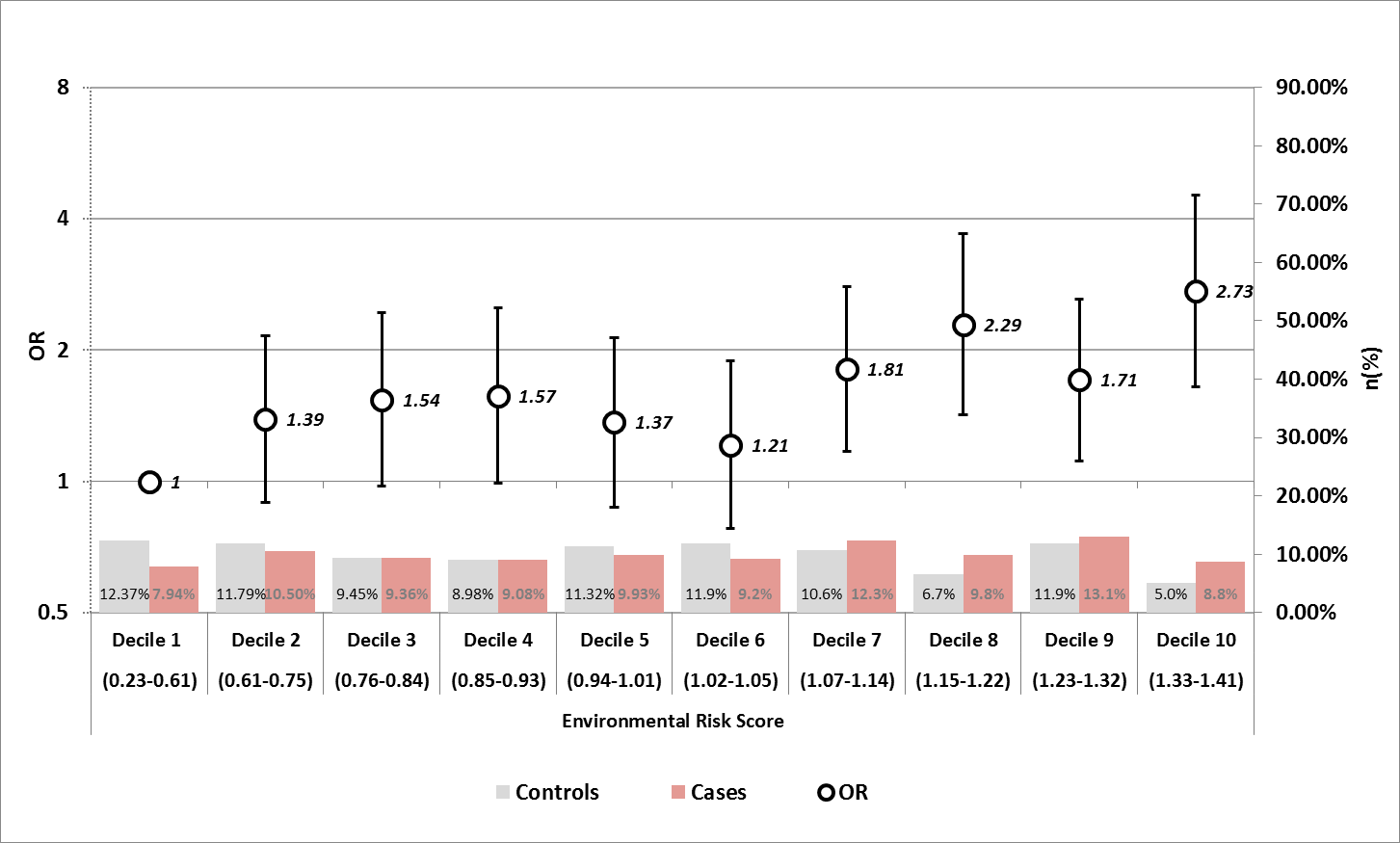

Supplement: Supplementary file 1 — Supplementary Information [file 41598_2017_9386_MOESM1_ESM.doc]
